# Tribute to Pasquale Montagna, 1950–2010

**DOI:** 10.1007/s10194-011-0330-8

**Published:** 2011-03-25

**Authors:** Pietro Cortelli

**Affiliations:** Clinica Neurologica, Dipartimento di Scienze Neurologiche, Alma Mater Studiorum, Università di Bologna, Via Ugo Foscolo, 7, 40123 Bologna, Italy

 Pasquale Montagna, MD, one of the most outstanding clinical neuroscientists and great leaders of the world of contemporary research on primary headache, died prematurely on December 9, 2010 at the age of 60 after courageously battling cancer for almost two and a half years. He never hid the fact that he was ill; he was aware of what was going to happen to him as he talked about it and tried to weigh up his life both in terms of achievements and failures. The example he set and the enthusiasm he conveyed over the years make us feel his absence so keenly.

Pasquale Montagna was born in a delightful small town in Muro Leccese in the southeastern part of Italy. He graduated in medicine from the University of Bologna in 1974 with a perfect score. Following his training in neurology at the University of Bologna, Montagna travelled to Copenhagen, Denmark to receive training in electromyography for 1 year at the famous Laboratory of Clinical Neurophysiology at Copenhagen University under the tutelage of Professor Fritz Buchthal. He later stayed there for 1 year following a fierce competition and winning the senior registrar position. In 1980 he became a researcher at the Bologna University Neurology Clinic, participating in teaching, research and patient care. In 1992 he won the public competition for Associate Professor, and in 2001 he became full Professor of Neurology at the Faculty of Medicine, Bologna University. Following Prof. Elio Lugaresi and Prof Agostino Baruzzi, Prof. Pasquale Montagna became Chairman of Neurological Sciences at the University of Bologna in 2007 and he remained in that position until his death.

Prof. Montagna was a member of a number of leading international Neurological Societies (American Neurological Association, American Academy of Neurology, Movement Disorders Society) and a member of the International Headache Society since 2000. In the field of headache research in Italy Pasquale Montagna was a founding member and Chairman of the Italian Association for the Study of Headache (ANIRCEF).

Pasquale Montagna’s career was replete with scientific and professional achievements in many fields of neurology including sleep medicine, movement disorders, clinical neurophysiology and headache. In particular, he and his colleagues discovered new clinical entities such as fatal familial insomnia, propriospinal myoclonus at sleep onset, nocturnal paroxysmal dystonia or nocturnal frontal lobe epilepsy, catathrenia, and periodic limb movements. In addition, Montagna was the first to postulate the hypothesis on the pathogenesis of migraine as an expression of dysregulation of brain oxidative metabolism [1].

Pasquale Montagna maintained his ambition to promote sleep medicine and headache research until the very end, proof-reading (as senior editor) the final chapters for the two volumes of Sleep Disorders (part of the Handbook of Clinical Neurology series) a couple of months before his sad demise and publishing a very important paper on the evolutionary meaning of primary headache in 2009, the second Darwin centenary [2]. Prof. Montagna contributed approximately 500 full papers in peer-reviewed and mostly international journals, over 227 presentations and abstracts and 59 book chapters.

The untimely death of Prof. Montagna at the height of his career remains an immeasurable loss but simply compiling his many major contributions does not convey a sense of the man. His collaborators, one and all, admired him for his great intellectual gifts and human warmth. They also feared the rigour of his judgement, but above all they loved him for what he had taught them, learnt more by example than by words. Reserved by nature, Montagna was modest and not one to talk about himself or his past or voice his opinion, being concerned more for the welfare of his patients and giving much of his time to his students, trainees and colleagues.

Like all great scientists, Montagna is survived by a rich legacy in headache and sleep research. At the time of his death, he was the undisputed leader of the Bologna group, whose sense of loss and grief has left an unfilled void. For me personally, Montagna was an invaluable mentor and his life work has helped us to progress in understanding brain function. There is no doubt that his prodigious contributions will be appreciated by the international neurological community for years to come.

Pasquale, my friend, although I am full of sadness, I live with pleasant memories, and I praise you for your passion for work, for constantly striving to help your patients, and for your unfailing devotion to your wife and young son. May your soul rest in peace. The headache community remains forever grateful to you for your lasting contributions. Your legacy, your fine example and teaching shall remain alive in the hearts and minds of all who had the privilege of living and working with you. Our compassion and prayers go to your wife, Flavia Valentini, and your son, Carlo Lorenzo, whose loss cannot be comprehended.

Pasquale Montagna remained optimistic until the very end. Just days before his death, looking out of the window he said “Pietro, look what a lovely sunny day it is. Let’s not spoil the mystery of nature that still has so much to reveal to us”. Then, with that look that defied reply he added “Work! Because doing good research is not just a pleasure, it is a duty you cannot avoid”.

Prof Pietro Cortelli

Clinica Neurologica

Dipartimento di Scienze Neurologiche

Alma Mater Studiorum, Università di Bologna

Via Ugo Foscolo, 7

40123 Bologna (Italy)

Email: pietro.cortelli@unibo.it
